# Salinity and Conductivity Amendment of Soil Enhanced the Bioelectrochemical Degradation of Petroleum Hydrocarbons

**DOI:** 10.1038/srep32861

**Published:** 2016-09-06

**Authors:** Xiaojing Li, Xin Wang, Yueyong Zhang, Qian Zhao, Binbin Yu, Yongtao Li, Qixing Zhou

**Affiliations:** 1 Agro-Environmental Protection Institute, Ministry of Agriculture, Tianjin 300191, China; 2 MOE Key Laboratory of Pollution Processes and Environmental Criteria/Tianjin Key Laboratory of Environmental Remediation and Pollution Control/College of Environmental Science and Engineering, Nankai University, Tianjin 300071, China; 3 College of Environmental Science and Engineering, Yangzhou University, Yangzhou 225127, China

## Abstract

The extreme salinity and high internal resistance of saline-alkali soil contaminated by petroleum hydrocarbons were two key limitations for using the bioelectrochemical remediation. In order to solve two problems, we simply rinsed soil, added carbon fiber to polluted soil. The charge output was enhanced by 110% with increase of the maximum current densities from 81 to 304 mA·m^−2^ while hydrocarbons degradation rate enhanced by 484%, especially the high molecular weight fractions (C28–C36 of *n*-alkanes and 4–6 rings of PAHs). These effects were possibly due to the selective enrichment of species belonged to δ*-Proteobacteria* (*Proteobacteria*), *Flavobacteriia* (*Bacteroidetes*) or *Clostridia* (*Firmicutes*), the activities of biological electron transfer and enzymes. As we know, oxygenase gene that directly decided the process of degradation, was surveyed for the first time in soil bioelectrochemical remediation system. The results confirmed that the bio-current stimulated the activities of naphthalene dioxygenase and xylene monooxygenase and thus the hydrocarbons degradation and the electricity generation. Given that electricity generation and the remediation performance are governed by multiple factors, understanding of microbial community and enzyme gene is crucial to promote the power yield and the bioelectrochemical remediation applicability.

Remediation of soils contaminated by petroleum hydrocarbons has been an arduous task, which attracts increasing attention since certain highly toxic and recalcitrant compounds are involved^1^,^2^. Physical and chemical remediation inevitably destroyed the original habitat and caused secondary pollution^3^,^4^. Though regular microbial technology and phytoremediation exert little effect on the pristine environment^5^,^6^, they are seriously restricted by ambient conditions (especially remote/extreme environment). Bioelectrochemical remediation, as an emerging technology, applies inexhaustible electrodes as the terminal electron acceptors to enhance biodegradation with the advantage of energy recovery^7^,^8^,^9^,^10^,^11^,^12^,^13^. Cost of remediation is greatly decreased as well as the scope is extended^10^,^14^,^15^. The soil microbial fuel cell (MFC) is one of commonly electrochemical biostimulation approaches and hardly exerts adverse influence on original soil structures and properties. Low cost, high efficiency and prevalent application are driving soil MFCs to be adopted widely.

Salt-influenced soils represent around 40% of the world’s lands^16^. Petroleum hydrocarbons contaminated saline-alkali soil is a common polluted environment because great oilfields always locate in coastal area. Due to the unfavorable high osmotic potential imposed by salts, the saline condition limits the activity of microorganism^17^,^18^. Qin *et al*. found that the degradation rate of petroleum hydrocarbons increased by about 30% when the soil salinity decreased from 2.86% to 0.10%^18^. Furthermore, high internal resistance is a key limiting factor to performance of MFCs^19^,^20^,^21^,^22^, particular in soil/sediment due to the low electrical conductivity^23^,^24^. It was noted that a 57% of decrement in internal resistance lead to a 100% of increment in current density in soil MFCs^25^. Additionally, our previous study found that U-type soil MFC with 7.4 Ω of internal resistance exhibited better performance of petroleum hydrocarbons degradation and power generation, compared to that with 42.6 Ω^10^.

Herein, two treatments including were conducted to enhance bioelectrochemical remediation of petroleum hydrocarbons contaminated saline-alkali soil. The performances of soil MFCs were investigated in terms of power generation, soil characteristics, hydrocarbons degradation, bacterial abundances. More importantly, oxygenase genes that have been widely used for the molecular monitoring of hydrocarbons biodegradation, were firstly quantified in soil MFCs. Simultaneously comprehensive correlations were surveyed to further understand the interaction among these factors in the bioeletrochemical remediation system.

## Results

### Performance of the soil MFC

**Figure 1:**
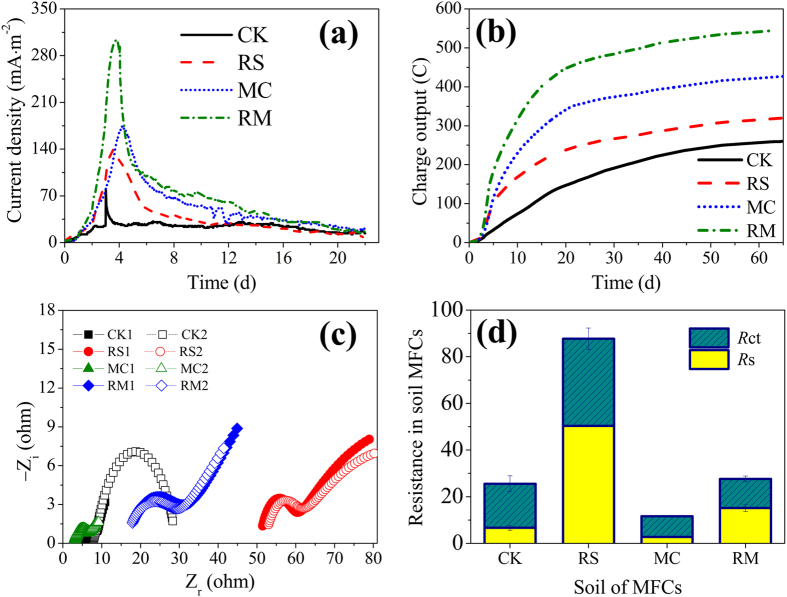
Current density during initial 22 days (**a**) and charge output during a period of 65 days (**b**) of the soil MFCs. The data is the mean value of duplicate soil MFCs (see Figure S1). Nyquist plots of the soil MFCs at open circuit potentials (**c**). The he resistance analysis of the soil MFCs (**d**). The charge transfer resistance *R*_ct_ = *R*_cathode_ + *R*_anode_.

The current densities of all treatments including RS, MC and RM were enhanced compared to CK (Fig. 1a and Figure S1a). The maximum current density of RS (135 ± 2 mA·m^−2^, averaged over 12 h of peak current) and MC (170 ± 2 mA·m^−2^, averaged over 12 h of peak current) were 214% and 295% higher than that of CK (43 ± 11 mA·m^−2^, averaged over 12 h of peak current). The peak of current density of RS was observed after 85 h, which was 16 h earlier than that of MC. Meanwhile, RS had the highest current density during initial 24 hours (Figure S2). For RM, 299 ± 4 mA·m^−2^ of maximum current density was acquired on day 4 and was 595%, 121% and 76% higher than CK, RS and MC respectively. The accumulated charge output of 260 ± 51 C for CK was gained during 65 days, which was 23%, 64% and 110% lower than these of RS (320 ± 82 C), MC (427 ± 91 C), and RM (546 ± 43 C) respectively (Fig. 1b and Figure S1b).

RS had the highest internal resistance of 88 ± 5 Ω, which was 238% higher than that of CK (26 ± 4 Ω), while the lowest internal resistance (12 ± 0 Ω) was observed in MC (Fig. 1c,d). There was no significant difference on total internal resistance between CK and RM (26 ± 2 Ω). For the soil samples rinsed salt (RS and RM), the Ohmic resistance (*R*_s_) increased. Particularly, *R*_s_ of RS (50 ± 0 Ω) enlarged by more than a factor of 6 compared to CK (7 ± 1 Ω). However, *R*_s_ of MC (3 ± 0 Ω) was only 43% of CK. Charge transfer resistances (*R*_ct_) exhibited a similar trend to the total internal resistances.

### Changes of soil characteristic

In the connected soil MFCs, soil pH decreased compared with original soil (OS, 8.30 ± 0.04) or rinsed salt original soil (RSOS, 9.08 ± 0.02) (Figure S3a). For CK and MC, only a 3–4% drop than OS was observed. While soil pH of RS and RM described a 10–13% lower than RSOS. For the connected MFCs, soil pH exhibited a slight differentiation. The soil electrical conductivities of RS (0.61 ± 0.01 mS∙cm^−1^) and RM (1.51 ± 0.00 mS∙cm^−1^) were 110% and 419% higher than that (0.29 ± 0.00 mS∙cm^−1^) of RSOS (Figure S3b). While CK (0.92 ± 0.00 mS∙cm^−1^) and MC (1.33 ± 0.00 mS∙cm^−1^) showed 49% and 27% of decline from OS (1.81 ± 0.04 mS∙cm^−1^). The soil electrical conductivities of connected soil MFCs showed a 14−49% of decrease from their corresponding controls. Both soil dehydrogenase and polyphenol oxidase activities in connected MFCs were much lower than those of OS or RSOS (Figure S3c,d). In addition, RS, MC or RM depicted lower enzyme activities than CK. Compared to the soil dehydrogenase, the soil polyphenol oxidase showed insignificant differences after bioelectrochemical remediation.

The soluble TC content of RSOS (419 ± 18 μg·g^−1^) was only 75% of the OS (560 ± 30 μg·g^−1^), which was partly accounted for the lower TC contents in rinsed salt soil samples (RS and RM) compared to CK and MC (Figure S4). Yet the soluble TN of RSOS (4.4 ± 0.3 μg·g^−1^) was comparable to the OS (4.8 ± 0.8 μg·g^−1^) indicating that nonionic nitrogen species were dominated in original soil. For all soil samples, TC and TN in connected MFCs showed 10–25% and 28–72% of reduction from the disconnected MFCs. Total organic carbon (TOC) was the main component of soluble TC in tested soil, and the dominant decrease of TC was inorganic carbon (IC) for CK and MC while TOC for RS and RM. Noticeably, the TN of soil in tested MFCs showed a evident increase (a factor of 16–70) from OS (or RSOS).

### Degradation of hydrocarbons

**Figure 2:**
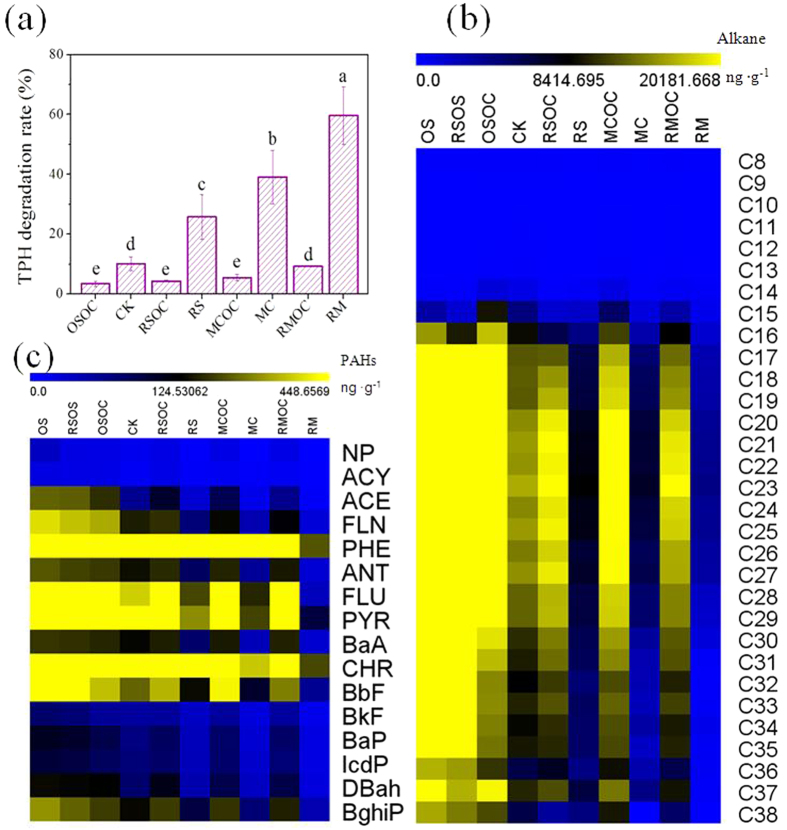
TPH degradation rates (**a**), concentrations of the *n*-alkanes (C8–C38) (**b**) and 16 priority PAHs (**c**) in soil of MFCs. The abbreviations of PAHs were noted in Figure S5. The different lowercase indicates significant difference at the 0.01 level (2-tailed).

As shown in Fig. 2a, the degradation rates of total petroleum hydrocarbons (TPHs) exhibited 153–484% of increment from CK in the connected MFCs after 65 days. Compared to their controls, the TPHs degradation rates in the connected MFCs were significantly (*p* < 0.01) higher and enhanced by a factor of 2–6. The highest degradation rate of TPHs was 60 ± 9% in RM, which was more than a factor of 20 as high as that in OSOC (3 ± 1%). The removal rates of TPHs were as follow: RM > MC > RS > CK.

Similar to TPHs, the contents of *n*-alkanes and PAHs were obviously reduced (Fig. 2b,c) and showed 59–92% and 44–88% of overall degradation rates in the connected MFCs (Figure S6). As expected, the concentration of *n*-alkanes and PAHs in the connected MFCs were much lower than those in the disconnected MFCs. In addition to the carbon fractions of C16–C19, the high molecular weight fractions (C28–C36) also showed an obvious reduction, particular in RM (Fig. 2b and Figure S6b). Generally the higher degradation rates were observed on low ring of PAHs (1–3 rings, C10–C13) over high ring PAHs (Fig. 2c and Figure S6d). However, even the high molecular weight of PAHs (4–6 rings, C16–C22) were degraded apparently after soil MFC remediation.

### Microbial community structures

According to the pyrosequencing of soil samples, the high-quality 16S rRNA gene 10492−20955 reads with an average length of 396 bp were constructed (Table S1). These sequences were assigned into 523−747 OTUs at a 97% similarity. Although new bacterial phylotypes continued to emerge even after 15,000 reads sampling with pyrosequencing, Shannon Index showed a plateau after 10,000 reads (Figure S7). This was also supported by 98–99% of Coverage estimators (Table S1), showing that the sizes of libraries were sufficient to cover the bacterial communities. The connected MFCs described the larger ACE and Chao 1 Index than those of the disconnected MFCs, while an inverse tendency was viewed for Shannon Index. Moreover, the highest ACE and Chao 1 estimators in RS indicated that its community had the greatest richness. The higher Shannon Index was observed in CK than others, showing that its biodiversity was most abundant.

**Figure 3:**
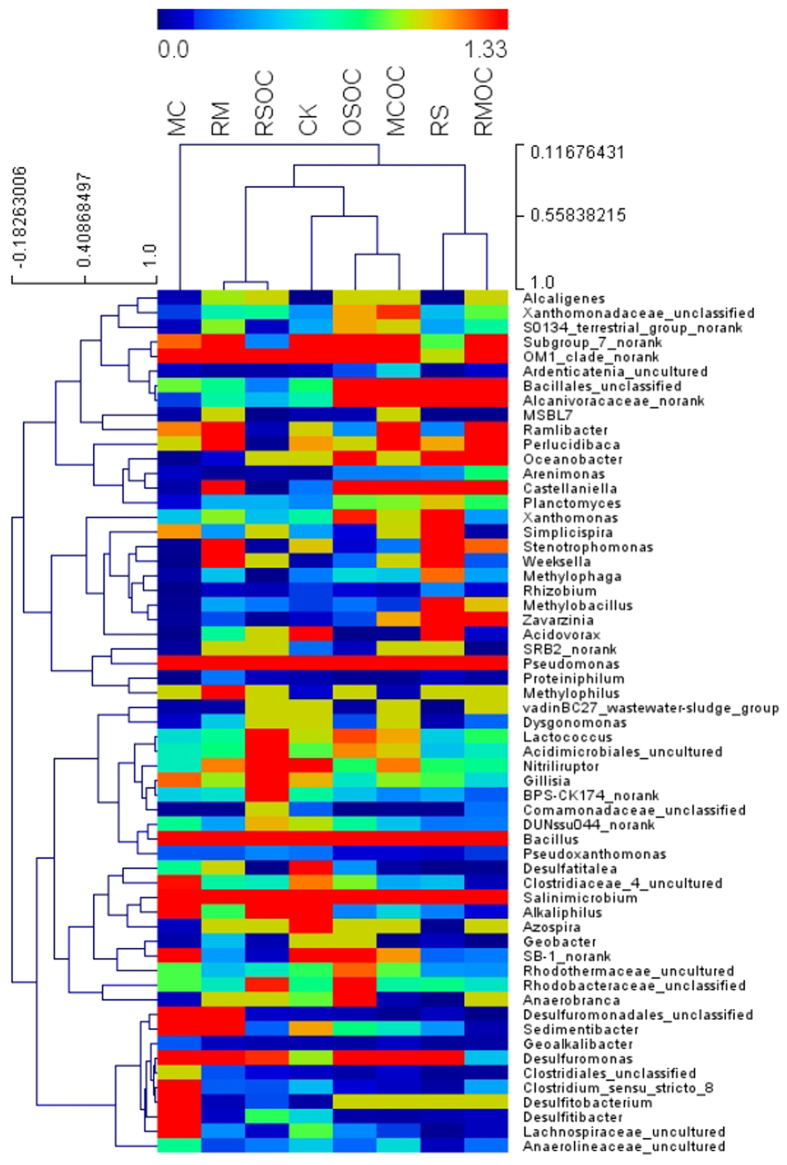
Taxonomic classification of bacterial DNA sequences from communities of soils in MFCs at the genus level. The genera that were less than 2% of total composition in all libraries were ignored in heat map.

Distinct cluster of bacterial communities were observed between rinsed and not rinsed salt soil samples, as well as between connected and disconnected MFCs from the hierarchical cluster analysis (Fig. 3). This was supported by principal component analysis (PCA) (Figure S8). Principal components 1 and 2 explained 40.32% and 25.62% of the total community variations, respectively. The sum of total observed OTUs in four communities of the connected MFCs was 1340, and 40% (537 OTUs) of the total OTUs were shared by them. OTUs of CK were the most (1073, 80% of total). The rest were as follow: RS (990, 74%) > MC (928, 69%) > RM (851, 64%), directly demonstrated that a selective enrichment was induced by bio-current.

**Figure 4:**
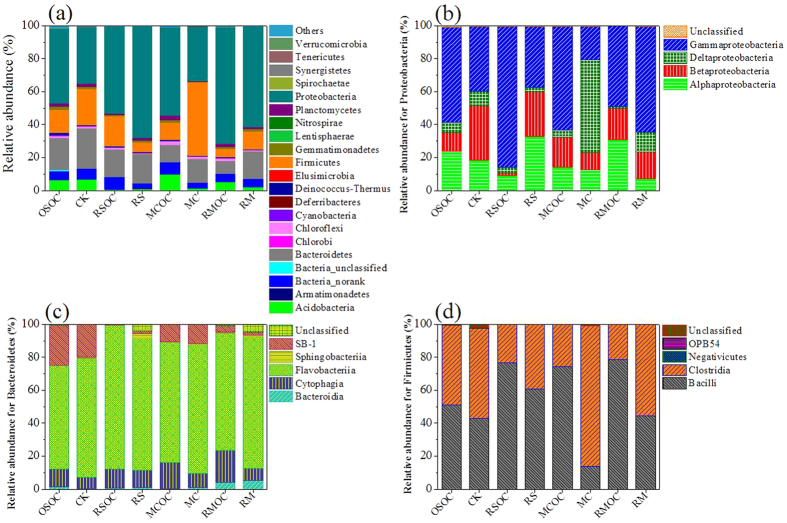
Taxonomic classification of bacterial DNA sequences from communities of soils in MFCs at the phylum level (**a**), the class level distribution of the most dominant phylum of *Proteobacteria* (**b**), and the following two dominant phyla, *Bacteroidetes* (**c**) and *Firmicutes* (**d**).

Twenty-two bacterial phyla were viewed from eight libraries (Fig. 4a). The absolute majority of sequences belonged to *Proteobacteria* that accounted for 34–71% of the total reads in bacterial community. *γ-Proteobacteria* was the most abundant class (20–85%) within the *Proteobacteria*, but its ratio reduced by 49–215% in connected MFCs compared to those in disconnected MFCs except RM (Fig. 4b). *α-Proteobacteria* abundance exhibited a similar trend to *γ-Proteobacteria*. In addition, the abundance of *β-Proteobacteria* rose in CK and RS, but declined in MC and RM. In comparison, an increase of composition was observed for *δ-Proteobacteria* in all connected MFCs compared with the corresponding disconnected MFCs.

Furthermore, the *Bacteroidetes*, *Firmicutes*, *Acidobacteria* and a certain amount of unclassified sequences (at the phylum level) were other major bacterial phyla. Compared to their controls, *Bacteroidetes* and *Firmicutes* abundances in connected MFCs increased while *Acidobacteria* abundance decreased in MC and RM. Within *Bacteroidetes*, the proportion of *Flavobacteriia* was the highest (Fig. 4c). *Bacteroidia* abundance described an increment, but a decrement for *Cytophagia*. Within *Firmicutes*, *Bacilli* and *Clostridia* were the majority (97.8–99.9%) in all soil MFCs (Fig. 4d). In connected MFCs, the ratio of *Bacilli* declined, but *Clostridia* rose.

The distribution of bacterial species at genus level was presented by a heat map (Fig. 3), which accounted for 70–84% of total composition in all libraries (genera that were less than 2% of total composition were ignored, Table S2). Similar to the results from Shannon Index, CK had the higher microbial diversity than any other. There were large number species of *Pseudomonas* (2.60−34.46%), *Salinimicrobium* (4.40–15.48%), *Bacillus* (1.33−11.64%) in all samples. The proportion of *Sedimentibacter* rose for all soil MFCs, showing a formative anaerobic environment. In CK, RS, MC and RM, the abundances of *Azospira*, *Geobacter* and *Desulfuromonas* significantly increased from their controls, particularly in RM. This increase of composition was also observed for *Desulfuromonadales* (unclassified), *Desulfitobacterium*, *Alkaliphilus*, *Castellaniella*. Abundance of *Clostridium* (unclassified) increased from 0.09% in MC to 10.35% in MCOC. In RS, *Alcanivoracaceae* as well as *Weeksella*, *Zavarzinia* and *Castellaniella* abundances showed some degree of increment, while *Bacillus* and *Pseudomonas* exhibited slight decrement compared to RSOC.

### Quantification of Oxygenase

**Figure 5:**
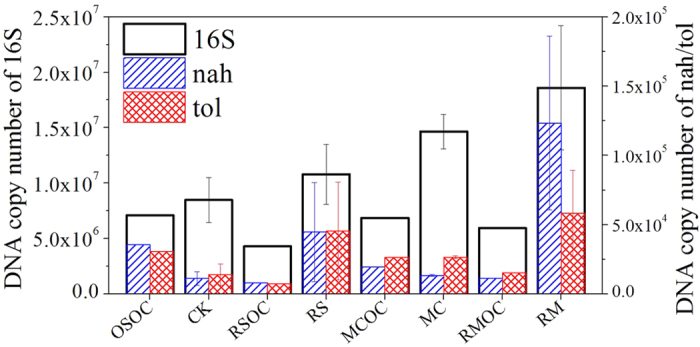
Real-time PCR quantification of total bacteria (16S), naphthalene dioxygenase (*nah*), xylene monooxygenase (*tol*) in different soil samples (ng^−1^ DNA).

Compared to the controls, the number of copies (ng^−1^ DNA) of total bacteria (16S) showed 20–214% of increase in connected MFCs (Fig. 5), due to the stimulation of bio-current. For the individual soil MFC, there was no apparent difference between investigated naphthalene dioxygenase (*nah*) and xylene monooxygenase (*tol*) gene copies (ng^−1^ DNA), and the total of both enzyme genes accounted for 0.27–0.98% of 16S. In RS and RM, copy numbers (ng^−1^ DNA) of *nah* and *tol* significantly increased from those in corresponding controls (a factor of 5–10 and 3–5 increase for *nah* and *tol* respectively). The most copy numbers (ng^−1^ DNA) of the total of *nah* and *tol* were observed in RM, which was a factor of 7 as many as that in CK.

## Discussion

The current generation of all soil MFCs peaked at around 4 days and then declined until the end of experiment, which was due to the depletion of easily biodegradable hydrocarbons^25^. Better performance (power production and hydrocarbons degradation) for RS and MC indicated that either salinity decrease or conductivity increase of soil had positive effect for MFC remediation in saline-alkali soil. After rinsed salt, the soluble salt content of soil decreased from 2.8% to 0.3% (Table S3) so that the suppress effect from salinity on the bacterial populations (especially exoelectrogen and degradation microbe) was relieved^18^,^26^. This was supported by higher current density during initial 24 h and shorter time of reaching peak current for RS. Furthermore, the internal resistance of soil blended with carbon fiber significantly declined especially *R*_ct_ (Fig. 1d), due probably to carbon fiber provided several conductive channels and thus promoted electron transfer and current collection, which enhanced the current density and remediation efficiency^23^. Simultaneously the best performance was detected when two amendments were combined (Figs 1 and 2). As an evidence, the power generation (current density or charge output) had obviously positive correlations (0.909–0.993**) with the degradation of petroleum hydrocarbons (TPH, Alkanes and PAHs) (Table 1), confirming that bioelectrochemical remediation of soil contaminated by hydrocarbons was a feasible approach with the advantage of energy recovery^7^,^9^,^12^,^13^. After rinsed salt, the soil osmotic pressure decreased, which was beneficial to the microbial activity. Yet the soil internal resistance significantly increased, which was adverse to the power production of soil MFC. Fortunately, the amendment treatment of blending carbon fiber completely overcame this defect. Consequently, the internal resistance of RM was comparable to that of CK (Fig. 1d). Compared to the Ohmic resistance (*R*_s_), the charge transfer resistance (*R*_ct_) had 154% and 323% more significantly negative correlations with the accumulated charge output and TPH degradation, indicating that the activity of biological electron transfer mainly determined the performance of bioelectrochemical remediation system^20^,^27^.

The statistical analysis showed that the soil pH showed a lower significant correlation with the TPH degradation or the charge output than the soil electrical conductivity (Table 1), indicating that the soil electrical conductivity is a vital property in soil bioelectrochemical remediation. Additionally, the significant correlations (−0.724*–−0.887**) between enzymatic activity and petroleum hydrocarbons degradation confirmed the biodegradation stimulated by bio-current was responsible for removal of hydrocarbons. Although the soil polyphenol oxidase activity showed a less decline from the original soil (Figure S3d), a more significant correlation was viewed between soil dehydrogenase activity and TPH degradation (−0.764*) or charge output (−0.839). Similar to previous reports^7^, these result also indicated that dehydrogenase activity may act as an bioindicator during the process of bioelectrochemical remediation.

Among abundances of top ten phyla, only *Proteobacteria*, *Firmicutes* and *Gemmatimonadetes* were positively correlated with the charge output (Table 1), indicating that a selective enrichment (e.g. exoelectrogen) was induced by bio-current^7^,^13^ and more importantly some species may grow by electron transfer or grow in association with specific members of the electrically active community^28^. As an evidence, γ- and δ-*Proteobacteria* that contained the common exoelectrogens (such as *Pseudomonas* and *Geobacter*^7^,^13^) showed the positive correlations with the electricity generation. Beside *Clostridia* and *Negativicutes* (Within *Firmicutes*), *Bacteroidia* abundances (within *Bacteroidetes*) were also positively correlated with the current density and charge output (0.965* and 0.902) (Table S4). These results suggested the certain distribution of electroactive microbes. Notably, only *Proteobacteria*, *Firmicutes* and *Bacterioidetes* abundances among top ten phyla had positively correlations to hydrocarbons degradation, indicated that the main hydrocarbons degraders belonged to them^29^,^30^,^31^. Significantly positive correlations between petroleum hydrocarbons degradations (TPH, alkane and PAH) and *Negativicutes* abundances (0.746*–0.803*) was observed (Table S4), confirmed that *Negativicutes* play a favorable role in hydrocarbon biodegradation^32^. Furthermore, similar correlations were viewed between petroleum hydrocarbons degradation and δ-*Proteobacteria* (0.494–0.543), *Bacilli* (0.404–0.586). Several species of these classes found to serve an effective in the removal of petroleum hydrocarbons^31^,^33^. Besides, *Planctomycetes* (−0.625), *Cyanobacteria* (−0.626), Bacteria_norank (−0.671), *Chloroflexi* (−0.671) closely related to *n*-alkanes degradation. These results suggested certain species (rather than all) of *Proteobacteria*, *Bacteroidetes* or *Firmicutes* played an affirmative role in degradation of hydrocarbons.

In addition to γ-*Proteobacteria* and *Bacilli*, the higher correlations between enzymatic activities and microbial abundances were observed in *Deinococcus-Thermus* and *Cyanobacteria*, nevertheless they were not the dominant hydrocarbons degraders or exoelectrogens, suggesting a subtle interaction existed between degraders and concomitant microbes. Furthermore, there were significantly positive correlations between *Deinococcus-Thermus* and *Cyanobacteria*, *Gemmatimonadetes* and *Cyanobacteria*, pointing out that a complex relationship occurred^34^. Furthermore, the charge transfer resistance described something with the special microbial community, such as 0.957* of correlation with α-*Proteobacteria*, 0.997* with *Sphingobacteriia*. The change of soil characteristics directly induced the evolution of community structure^35^. For example, increase of soil pH clearly reduced the abundance of *Acidobacteria* (Fig. 4). Additionally, soil pH had a distinctly positive correlation (0.707) with abundance of γ-*Proteobacteria* that involved in most reported exoelectrogens^7^. Moreover, there were significant correlation between soil electrical conductivity and abundance of *Bacilli* (0.715*), between soil polyphenol oxidase and abundances of *Negativicutes* (−0.753*), between soil dehydrogenase and *Deinococcus*-Thermus (0.733*) (Table 1 and Table S4), which need to be further addressed in the succedent plan.

In bioelectrochemical remediation system, copies of naphthalene dioxygenase (*nah*), xylene monooxygenase (*tol*) exhibited a high correlation (0.899 and 0.827) with current density of soil MFCs (Table 2). Compared to *nah*, copies of *tol* had a more distinct correlation with hydrocarbon degradation (alkane and PAH). By contrast, copies of total bacteria populations (16S) obviously positively related to hydrocarbon degradation (0.896**–0.975**) and electricity generation (0.972**–1.000**), implying that biodegradable hydrocarbons was the strong determinant of total bacterial abundance and power output^26^. Previous reports showed that amount of *nah* and *tol* could use as bioindicator in degradation of petroleum hydrocarbons contaminated soil^36^. In soil MFCs, it was also demonstrated that the quantitative determination of key enzyme gene was feasible as a monitoring or predicting index during pollution remediation.

## Methods

### Tested soil and treatment

Petroleum hydrocarbon contaminated soil was collected from the topsoil (<10 cm) near the oil well in Dagang Oilfield of Binhai New Area (Tianjin, China). The soil was air-dried, passed 2 mm sieve and marked as original soil (OS). The soil properties were summarized as Table S3. The OS was rinsed with 1:1.2 (*w*/*v*) of distilled water by water leaching method^18^ and marked as RSOS. The connected MFCs filled with OS and RSOS were marked as CK and RS, while the corresponding disconnected controls as OSOC and RSOC. The connected MFC filled with the mixture of OS and carbon fiber pieces (1 cm of length, 5 μm of diameter, Jilin Carbon Factory; acetone clean overnight; 2% in mass ratio to soil) was marked as MC and the corresponding disconnected control as MCOC. The MFC filled with soil that was rinsed salt and followed by blended of carbon fiber (2%), was marked as RM and its disconnected control as RMOC. The abbreviations used in this study were shown in Table 3. All soil MFCs were filled with 100 g of dry soil without adding buffer solution or any exogenetic inoculation.

### Soil MFC configuration

Soil MFCs were constructed as previous describe^13^ with only one layer of anode as showed in Figure S9. Carbon mesh (Jilin Carbon Factory, Jilin, China) was used as the anode after acetone clean overnight^37^. Activated carbon air-cathode was prepared according to the modified rolling-press method^19^,^20^. All soil MFCs were placed in an incubator (30 °C) and sealed with distilled water during the experiment. Each treatment including their disconnected control had a duplicate.

### Electrochemical analysis

Voltage (*U*) across the external resistor of 100 Ω (*R*) was recorded by a data acquisition system (PISO-813, ICP DAS Co., Ltd, Shanghai, China). A potentiostat (Autolab PGSTAT 302N, Metrohm, Switzerland) was used to perform electrochemical impedance spectrum (EIS) from 100 kHz to 1 Hz with a 10 mV of amplitude at the open circuit potential (stabilized for 4 h after installed). The carbon mesh anode was used as the working electrode while the air-cathode as the counter and reference electrode. ZsimpWin 3.10 was employed to simulate Nyquist plots according to an equivalent circuit in Figure S10.

### Chemical analysis

The soil pH and the electrical conductivity were measured in a mixture with soil to distilled water ratio (*w*/*v*) as 1:5. Organic matter, available N, P and K were evaluated according to regular methods^38^. The soluble salt content was measured as previous description^18^. The contents of metal elements were attained using ICP-OES (Vista MPX Varian, US)^39^. The soil dehydrogenase was extracted by 2,3,5-Triphenyl Tetrazolium Chloride reduction and Triphenyl Formazone calibration (485 nm). Soil polyphenol oxidase was analyzed by catalyzing pyrogallol (430 nm) as previous methods^40^. The soluble total carbon (TC) and total nitrogen (TN) were determined by an Analytik jena multi N/C 3100 (Germany) (Details found in the supporting information). The total petroleum hydrocarbon (TPH) and carbon fractions (*n*-alkanes and PAHs) were analyzed according to our previous procedures^6^,^10^.

### Biological analysis

Genomic DNA was extracted using a Power Soil DNA Isolation Kit (Mobio, USA) according to the manufacturer’s instruction. 1% agarose gel electrophoresis was employed to confirm the purity of genomic DNA.

Bacterial 16S rRNA gene fragments were PCR-amplified using primers of 515F (5′-GTGCCAGCMGCCGCGG-3′) and 907R (5′-CCGTCAATTCMTTTRAGTTT-3′) covering V4 and V5 regions. PCR was carried out in triplicate 20 μL reactions containing 4 μL of 5× FastPfu Buffer, 2 μL of 2.5 mM dNTPs, 0.8 μL of each Primer (5 μM), 0.4 μL of FastPfu Polymerase, 10 ng of Template DNA, and added ddH_2_O to bring up the final volume to 20 μL. The PCR temperature procedures were 95 °C for 3 min; 25 cycles consisted of 95 °C for 30 s, 55 °C for 30 s and 72 °C for 45 s; 72 °C for 10 min and 4 °C until halted. After confirmed by 2% agarose gel electrophoresis, PCR products were pooled and purified using AxyPrepDNA (Axygen, USA).

Pyrosequencing was performed on a Genome Sequencer instrument (MiSeq) as the standard procedure of Majorbio Bio-Pharm Biotechnology Co., Ltd. (Shanghai, China). Quality filters were used to remove low quality sequences which were shorter than 50 bp, involved in any ambiguous bases, showed a homopolymer of longer than 10 bp, or were chimera. Finally, the valid sequences were trimmed to an average length of 396 bp. Then these sequences were clustered into Operational Taxonomic Units (OTUs) based on a 97% similarity threshold. Ribosomal Database Project classifier was used to align for their taxonomy at different levels (http://www.arb-silva.de).

The quantitative determination of naphthalene dioxygenase (*nah*), xylene monooxygenase (*tol*) and total bacteria populations (16S) were determined by ABI7500 FAST system with the primers^41^ listed in Table S5. A 25 μL real-time PCR mixture contained 12.5 μL of 2× SybrGreen qPCR Master Mix, 0.5 μL of each primer (10 μM), 1 μL of Template DNA and 10.5 μL of ddH_2_O. PCR conditions were as follow: initial denaturation at 95 °C for 2 min; 40 cycles consisted of 95 °C for 10 s and 60 °C for 40 s.

### Calculation

The current density (mA·m^−2^) of MFC was obtained as *I*´ = *U*/(*R*·*A*), where *A* is the area of air-cathode (0.0036 m^2^). The charge output was evaluated as *Q* = ∫_0_^*T*^(*U*/*R*)d*t*. The degradation rate of hydrocarbons was gained as *η* = (*C*_OS_ – *C*)/*C*_OS_, where *C*_OS_ is the hydrocarbons concentration in original soil (OS) or rinsed salt original soil (RSOS) as shown in Figure S10 and *C* is the hydrocarbons concentration in tested soil. The overall degradation rate of PAHs (or *n*-alkanes) was calculated by adding the contents of 16 PAHs (or 31 kinds of *n*-alkanes). The community richness and diversity estimates (Rarefaction, Chao 1, ACE and Shannon Index) were calculated according to the assigned methods (http://www.mothur.org/wiki/). Relative abundance was defined as that the number of sequences affiliated with that phylum, genus, or class divided by the total sequence number of per sample or dominant phyla (*Proteobacteria*, *Bacteroidetes* or *Firmicutes*).

## Additional Information

**How to cite this article**: Li, X. *et al*. Salinity and Conductivity Amendment of Soil Enhanced the Bioelectrochemical Degradation of Petroleum Hydrocarbons. *Sci. Rep.* **6**, 32861; doi: 10.1038/srep32861 (2016).

## Supplementary Material

Supplementary Information

## Figures and Tables

**Table 1:** Pearson correlation matrix (2-tailed) between dominant phyla and parameters of soil MFCs.

Index	*I*	*Q*	*R*s	*Rct*	pH	EC	DHA	PPO	TPH	Alkane	PAH
*I*^a^	1										
*Q*^a^	0.976*	1									
	0.024										
*R*s^a^	−0.081	−0.226	1								
	0.919	0.774									
*R*ct^a^	−0.433	−0.574	0.925	1							
	0.567	0.426	0.075								
pH^b^	0.338	0.23	−0.32	−0.298	1						
	0.662	0.770	0.680	0.702							
EC^b^	0.592	0.533	0.663	0.335	0.162	1					
	0.408	0.467	0.337	0.665	0.702						
DHA^b^	−0.839	−0.76	−0.461	−0.096	0.111	0.334	1				
	0.161	0.240	0.539	0.904	0.793	0.419					
PPO^b^	−0.891	−0.967*	0.332	0.664	0.17	0.063	0.765*	1			
	0.109	0.033	0.668	0.336	0.687	0.882	0.027				
TPH^b^	0.982*	0.993**	−0.113	−0.478	−0.004	−0.403	−0.764*	−0.788*	1		
	0.018	0.007	0.887	0.522	0.992	0.323	0.027	0.020			
Alkane^b^	0.909	0.945	−0.016	−0.393	−0.063	−0.514	−0.887**	−0.724*	0.930**	1	
	0.091	0.055	0.984	0.607	0.882	0.192	0.003	0.042	0.001		
PAH^b^	0.919	0.950*	−0.013	−0.391	−0.211	−0.535	−0.867**	−0.772*	0.940**	0.966**	1
	0.081	0.050	0.987	0.609	0.616	0.172	0.005	0.025	0.001	0.000	
*Pro*^b^	0.451	0.282	0.833	0.607	0.106	0.387	−0.294	−0.093	0.07	0.053	−0.013
	0.549	0.718	0.167	0.393	0.803	0.344	0.480	0.827	0.870	0.901	0.976
*Bac*^b^	−0.632	−0.718	−0.091	0.241	0.196	−0.578	0.172	0.432	0.04	0.062	0.101
	0.368	0.282	0.909	0.759	0.642	0.133	0.684	0.286	0.926	0.884	0.812
*Fir*^b^	−0.192	0.025	−0.737	−0.669	−0.196	−0.356	0.009	−0.204	0.229	0.29	0.28
	0.808	0.975	0.263	0.331	0.643	0.386	0.983	0.627	0.585	0.486	0.501
*Nor*^b^	−0.14	−0.206	−0.458	−0.252	0.621	0.556	0.553	0.362	−0.572	−0.671	−0.681
	0.860	0.794	0.542	0.748	0.100	0.152	0.155	0.378	0.139	0.068	0.063
*Aci*^b^	−0.55	−0.58	−0.419	−0.091	−0.164	0.156	0.282	0.11	−0.54	−0.58	−0.431
	0.450	0.420	0.581	0.909	0.697	0.712	0.498	0.795	0.167	0.132	0.286
*Chl*^b^	−0.46	−0.306	−0.845	−0.6	−0.095	0.454	0.485	0.089	−0.627	−0.671	-0.595
	0.540	0.694	0.155	0.400	0.823	0.258	0.223	0.833	0.096	0.068	0.120
*Gem*^b^	0.535	0.378	0.016	−0.074	0.033	0.272	0.331	0.052	−0.222	−0.487	−0.318
	0.465	0.622	0.984	0.926	0.938	0.515	0.423	0.904	0.597	0.221	0.442
*Pla*^b^	−0.415	−0.596	0.445	0.648	−0.168	0.352	0.245	0.209	−0.584	−0.625	−0.503
	0.585	0.404	0.555	0.352	0.690	0.392	0.558	0.619	0.129	0.098	0.204
*Cya*^b^	−0.115	−0.32	0.859	0.874	−0.341	0.098	0.627	0.447	−0.426	−0.626	−0.46
	0.885	0.680	0.141	0.126	0.408	0.818	0.096	0.266	0.292	0.097	0.251
*Dei*^b^	−0.567	−0.402	−0.747	−0.491	−0.36	−0.164	0.733*	0.446	−0.416	−0.57	−0.427
	0.433	0.598	0.253	0.509	0.381	0.699	0.038	0.268	0.305	0.141	0.292

*I*, the maximum current density; *Q*, charge output; *R*s and *R*ct represent Ohmic and charge transfer resistance; EC, electrical conductivity; DHA, dehydrogenase; PPO, polyphenol oxidase; TPH, Alkane and PAH indicate degradation rates of total petroleum hydrocarbon, *n*-alkanes and PAHs; *Pro*, *Bac*, *Fir*, *Nor*, *Aci*, *Chl*, *Gem*, *Pla*, *Cya*, *Dei* denote *Proteobacteria*, *Bacteroidetes*, *Firmicutes*, *Bacteria*_norank, *Acidobacteria*, *Chloroflexi*, *Gemmatimonadetes*, *Planctomycetes*, *Cyanobacteria*, *Deinococcus*-Thermus respectively, which were top ten abundances at phylum level.

^a^n = 4.

^b^n = 8.

^*^Correlation is significant at the 0.05 level (2-tailed).

^**^Correlation is significant at the 0.01 level (2-tailed).

**Table 2:** Pearson correlation matrix (2-tailed) between gene copies and parameters of soil MFCs.

Index	*I*^a^	*Q*^a^	*R*_*s*_^a^	*R*_*ct*_^a^	pH^b^	EC^b^	DHA^b^	PPO^b^	TPH^b^	Alkane^b^	PAH^b^	*16S*	*nah*	*tol*
*16S*^b^	0.972*	1.000**	−0.219	−0.570	−0.119	−0.495	−0.725*	−0.776*	0.975**	0.896**	0.954**	1		
	0.028	0.000	0.781	0.430	0.778	0.213	0.042	0.023	0.000	0.003	0.000			
*nah*^b^	0.899	0.784	0.175	−0.123	0.198	−0.173	−0.482	−0.523	0.775*	0.562	0.629	0.760*	1	
	0.101	0.216	0.825	0.877	0.639	0.682	0.227	0.184	0.024	0.147	0.095	0.029		
*tol*^b^	0.827	0.717	0.491	0.148	−0.229	−0.197	−0.573	−0.565	0.762*	0.609	0.731*	0.789*	0.892**	1
	0.173	0.283	0.509	0.852	0.585	0.640	0.138	0.144	0.028	0.109	0.039	0.020	0.003	

16S, total bacteria populations; *nah*, naphthalene dioxygenase; *tol*, xylene monooxygenase.

^a^n = 4.

^b^n = 8.

^*^Correlation is significant at the 0.05 level (2-tailed).

^**^Correlation is significant at the 0.01 level (2-tailed).

**Table 3:** Abbreviations of soil samples used in this study.

Sample ID	Sample source
OS	Original soil
RSOS	Original soil via rinsed salt
OSOC	Disconnected MFC filled with OS
CK	Connected MFC filled with OS
RSOC	Disconnected MFC filled with RSOS
RS	Connected MFC filled with RSOS
MCOC	Disconnected MFC filled with OS mixed with carbon fiber
MC	Connected MFC filled with OS mixed with carbon fiber
RMOC	Disconnected MFC filled with RSOS mixed with carbon fiber
RM	Connected MFC filled with RSOS mixed with carbon fiber
