# Supplementary material for: Salinity and Conductivity Amendment of Soil Enhanced the Bioelectrochemical Degradation of Petroleum Hydrocarbons
Source: Sci Rep. 2016 Sep 6;6:32861. doi: 10.1038/srep32861 (PMC5011858; doi:10.1038/srep32861)
Supplement: Supplementary Information [file srep32861-s1.pdf]

–Supplementary Information–

Salinity and Conductivity Amendment of Soil Enhanced the Bioelectrochemical Degradation of Petroleum Hydrocarbons

Xiaojing Li [a,b], Xin Wang [b,*], Yueyong Zhang [b], Qian Zhao [b], Binbin Yu [c], Yongtao Li [a], Qixing Zhou [b,*]

[a] Agro-Environmental Protection Institute, Ministry of Agriculture, Tianjin 300191, China

[b] MOE Key Laboratory of Pollution Processes and Environmental Criteria / Tianjin Key Laboratory of Environmental Remediation and Pollution Control / College of Environmental Science and Engineering, Nankai University, Tianjin 300071, China

[c] College of Environmental Science and Engineering, Yangzhou University, Yangzhou 225127, China

*Corresponding Authors: Phone: (86)22-23611021; fax: (86)22-23613820.

lixiaojing@caas.cn (Li X.);

xinwang1@nankai.edu.cn (Wang X.);

859701416@qq.com (Zhang Y.)

zqtalent@163.com (Zhao Q.)

bbyu@yzu.edu.cn (Yu B.)

yongtao@scau.edu.cn (Li Y.)

zhouqx@nankai.edu.cn (Zhou Q.)

Analysis of the soluble total carbon (TC) and total nitrogen (TN)

Briefly, 4 g of the soil was blended with 20 mL of distilled water. This homogenized slurry was shaken at 180 rpm for 24 hours and subsequently centrifuged at 4000 rpm for 5 min. Prior to the analysis, the supernatant was filtered through a 0.45 μm filter membrane.

Table S1 97% Similarity-based OTUs and species richness and diversity estimates

Sample	Reads	0.97				
		OTU	ACE	Chao 1	Shannon	Coverage
OSOC	18482	729	901	902	4.74	0.9896
CK	17583	747	945	936	4.68	0.9881
RSOC	20955	684	934	957	3.79	0.9892
RS	19724	656	1078	978	4.26	0.9885
MCOC	20631	671	856	827	4.28	0.9911
MC	10634	523	960	828	4.15	0.9803
RMOC	11318	532	727	729	4.20	0.9842
RM	11486	588	815	825	4.17	0.9820
Total	130813	5130	7216	6982		

Table S2 Genus resolution (%) when the genera that were less than 2% of total composition in all libraries were ignored.

OSOC	CK	RSOC	RS	MCOC	MC	RMOC	RM
70	74	81	77	78	84	76	81

Table S3 Properties of petroleum hydrocarbons contaminated soil, unit: mg kg^{-1}

Index	Value
pH	8.30
conductivity (mS cm^{-1})	1.81
soluble salt content	2.83%
soluble salt content after rinsed salt	0.33%
available nitrogen	46
available phosphorus	198
available potassium	525
organic matter	142
Zn	54
Cu	9
Ni	14
Mn	58
Fe	3101
Pb	19
Cr	60
Cd	ND

Table S4 Pearson correlation matrix (2-tailed) between dominant classes and parameters of soil MFCs.

Index	I^a	Q^a	$R_s{}^a$	$R_{ct}{}^a$	pH[b]	EC[b]	DHA[b]	PPO[b]	TPH[b]	Alkan[b]e	PAH[b]
α-Pro[b]	-0.624	-0.714	0.826	0.957*	-0.515	-0.184	0.000	0.383	-0.354	-0.19	-0.179
	0.376	0.286	0.174	0.043	0.192	0.662	1.000	0.348	0.390	0.652	0.672
β-Pro[b]	-0.682	-0.817	0.361	0.651	-0.205	-0.553	-0.473	-0.005	0.033	0.236	0.299
	0.318	0.183	0.639	0.349	0.627	0.155	0.236	0.990	0.938	0.573	0.472
γ-Pro[b]	0.652	0.494	0.113	-0.042	0.707	0.704	0.469	0.278	-0.291	-0.499	-0.551
	0.348	0.506	0.887	0.958	0.050	0.051	0.242	0.504	0.485	0.208	0.157
δ-Pro[b]	0.087	0.301	-0.605	-0.672	-0.392	-0.379	-0.266	-0.511	0.494	0.528	0.543
	0.913	0.699	0.395	0.328	0.337	0.355	0.525	0.196	0.213	0.178	0.164
b-Bac[b]	0.965*	0.902	-0.099	-0.404	0.245	-0.183	-0.425	-0.498	0.586	0.443	0.403
	0.035	0.098	0.901	0.596	0.559	0.664	0.293	0.209	0.127	0.272	0.322
c-Bac[b]	-0.184	-0.228	0.867	0.783	-0.127	0.602	0.166	0.075	-0.523	-0.494	-0.535
	0.816	0.772	0.133	0.217	0.765	0.114	0.694	0.859	0.184	0.213	0.172
f-Bac[b]	0.689	0.682	0.444	0.085	0.442	0.246	-0.366	-0.229	0.392	0.47	0.302
	0.311	0.318	0.556	0.915	0.272	0.557	0.372	0.585	0.337	0.240	0.467
s-Bac[b]	-0.364	-0.515	0.945	0.997**	-0.231	-0.275	-0.51	0.097	0.131	0.348	0.327
	0.636	0.485	0.055	0.003	0.581	0.510	0.197	0.820	0.756	0.399	0.430
b-Fir[b]	-0.073	-0.284	0.807	0.822	0.343	0.715*	0.283	0.391	-0.589	-0.59	-0.665
	0.927	0.716	0.193	0.178	0.405	0.046	0.496	0.338	0.124	0.124	0.072
c-Fir[b]	0.108	0.32	-0.786	-0.82	-0.353	-0.705	-0.289	-0.404	0.603	0.598	0.674
	0.892	0.680	0.193	0.180	0.391	0.051	0.488	0.320	0.114	0.117	0.067
n-Fir[b]	0.578	0.742	-0.57	-0.796	-0.19	-0.355	-0.484	-0.753*	0.803*	0.746*	0.756*
	0.422	0.258	0.430	0.204	0.650	0.388	0.224	0.031	0.016	0.034	0.030
o-Fir[b]	-0.67	-0.68	-0.405	-0.049	-0.046	-0.416	0.481	0.501	-0.436	-0.449	-0.322
	0.330	0.320	0.595	0.951	0.915	0.306	0.228	0.205	0.280	0.265	0.436

I, the maximum current density; *Q*, charge output; *R*s and *R*ct represent Ohmic and charge transfer resistance; EC, electricity conductivity; DHA, dehydrogenase; PPO, polyphenol oxidase; TPH, Alkane and PAH indicate degradation rates of total petroleum hydrocarbon, *n*-alkanes and PAH; α-*Pro*, β-*Pro*, γ-*Pro*, δ-*Pro*, b-*Bac*, c-*Bac*, f-*Bac*, s-*Bac*, b-*Fir*, c-*Fir*, n-*Fir*, o-*Fir* denote α-*Proteobacteria*, β-*Proteobacteria*, γ-*Proteobacteria*, δ-*Proteobacteria, Bacteroidia, Cytophagia, Flavobacteriia, Sphingobacteriia, Bacilli, Clostridia, Negativicutes*, OPB54, respectively.

[a] n = 4; [b] n = 8

*, correlation is significant at the 0.05 level (2-tailed); **, correlation is significant at the 0.01 level (2-tailed).

Table S5 The primers that were used in the microbial experiments.

Target group	Primer	Sequence	Length
16S	Eub338	5′-ACTCCTACGGGAGGCAGCAG-3′	181
	Eub518	5′-ATTACCGCGGCTGCTGG-3′	
nah	nah-F	5′-CAAAARCACCTGATTYATGG-3′	376
	nah-R	5′-AYRCGRGSGACTTCTTTCAA-3′	
tol	tol-F	5′-TGAGGCTGAAACTTTACGTAGA-3′	475
	tol-R	5′-CTCACCTGGAGTTGCGTAC-3′	

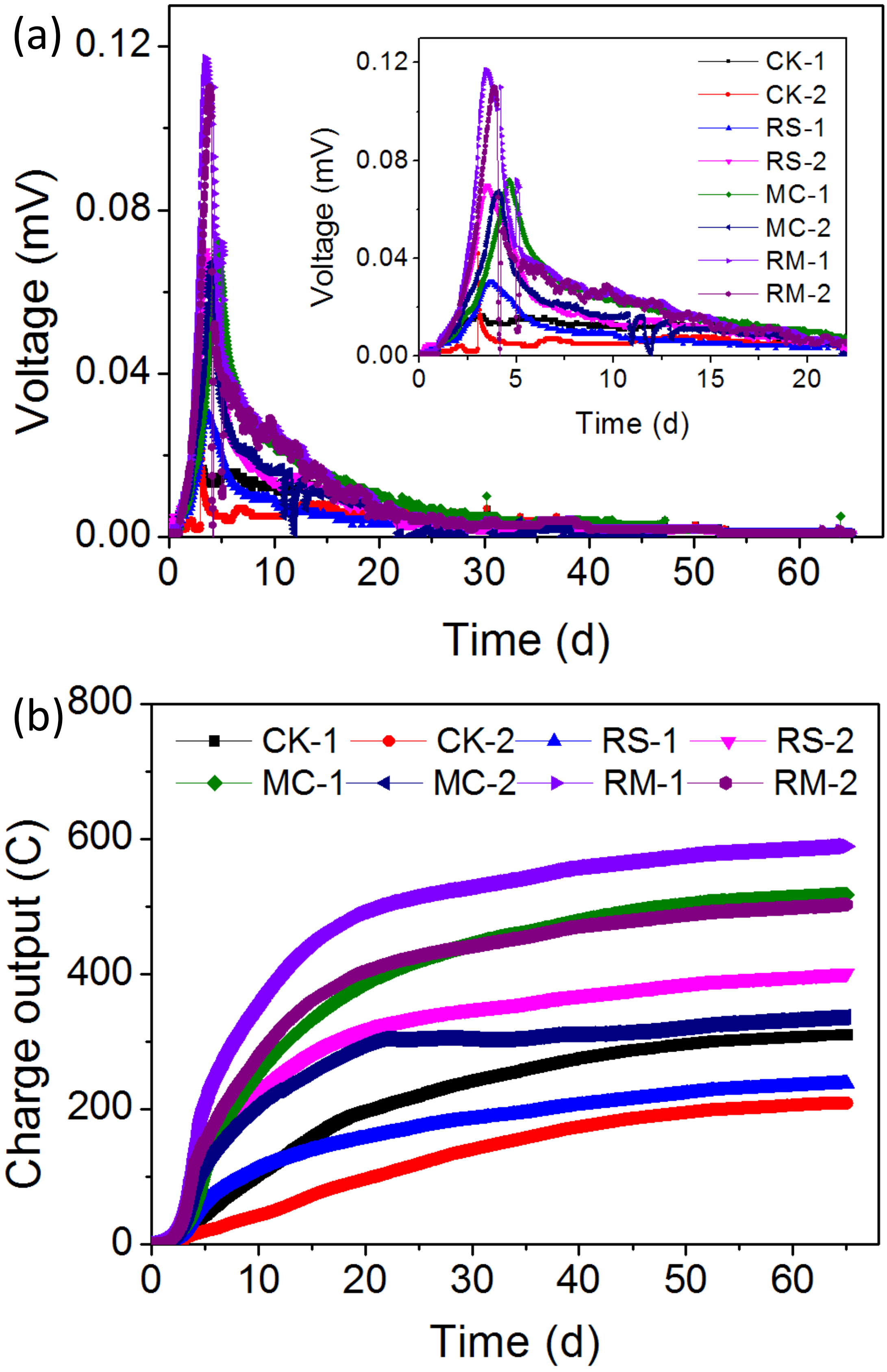

Figure S1 Voltage generation (a) and charge output (b) of soil MFCs. The inserted figure is the voltage generation of the soil MFC during initial 22 days.

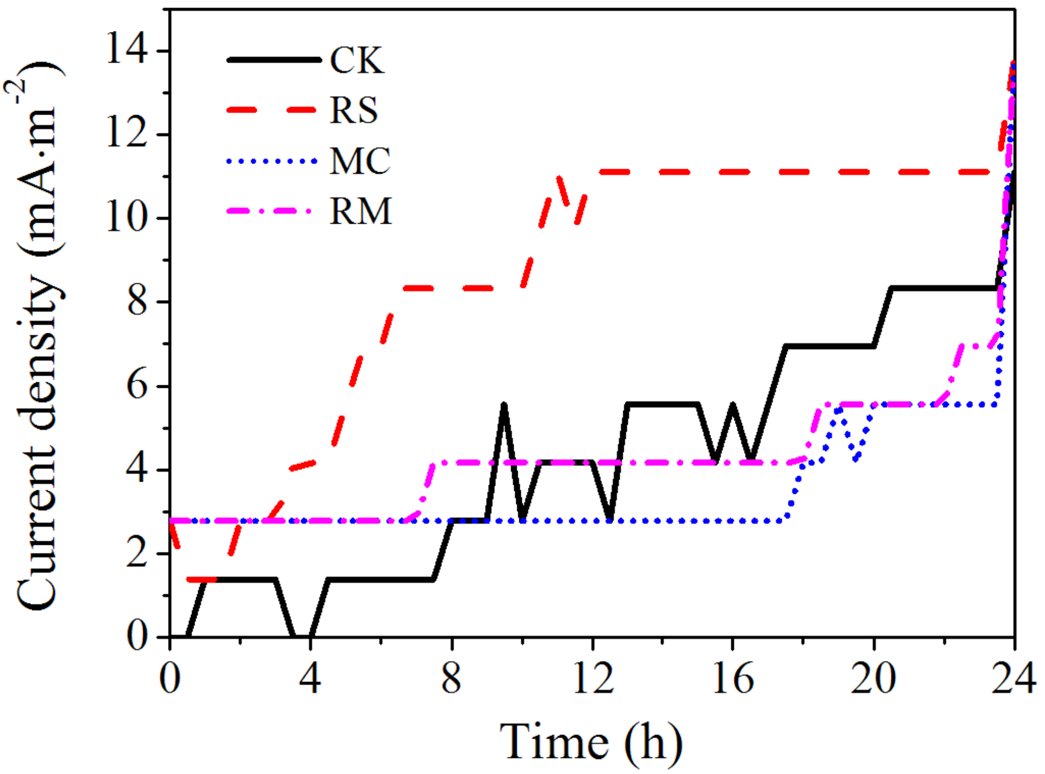

Figure S2 Current density of the soil MFCs during initial 24 hours.

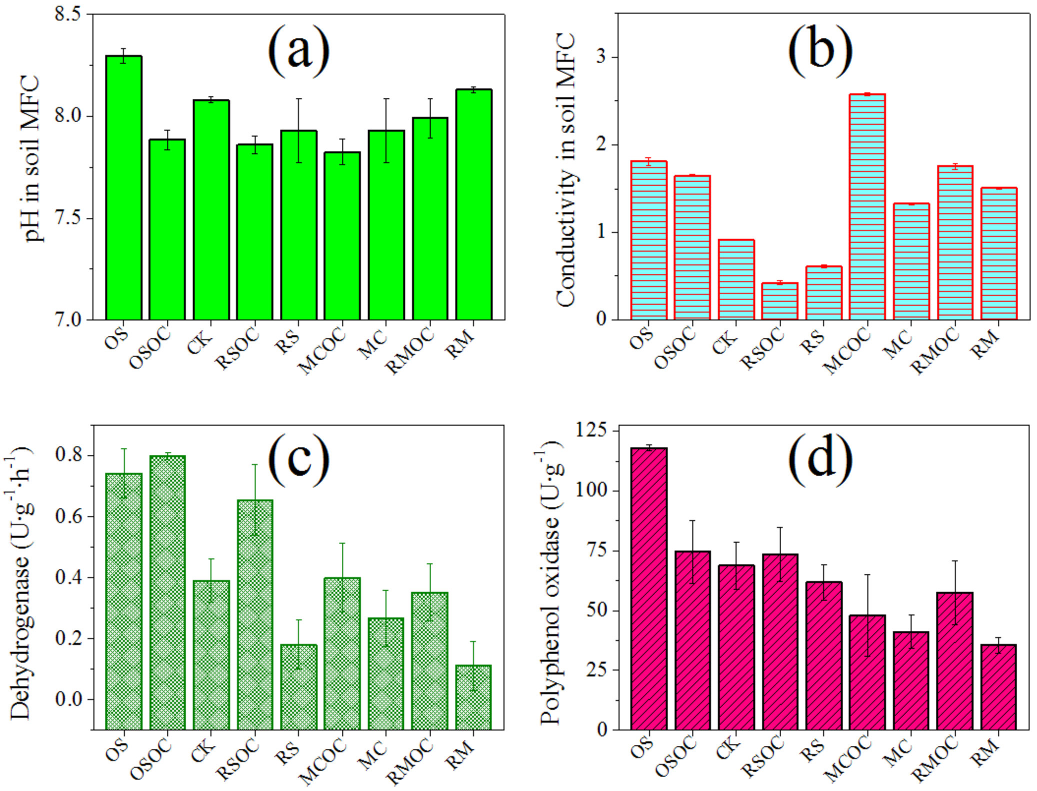

Figure S3 Soil pH (a), electrical conductivity (b), dehydrogenase (c) and polyphenol oxidase activities (d) of the soil MFCs.

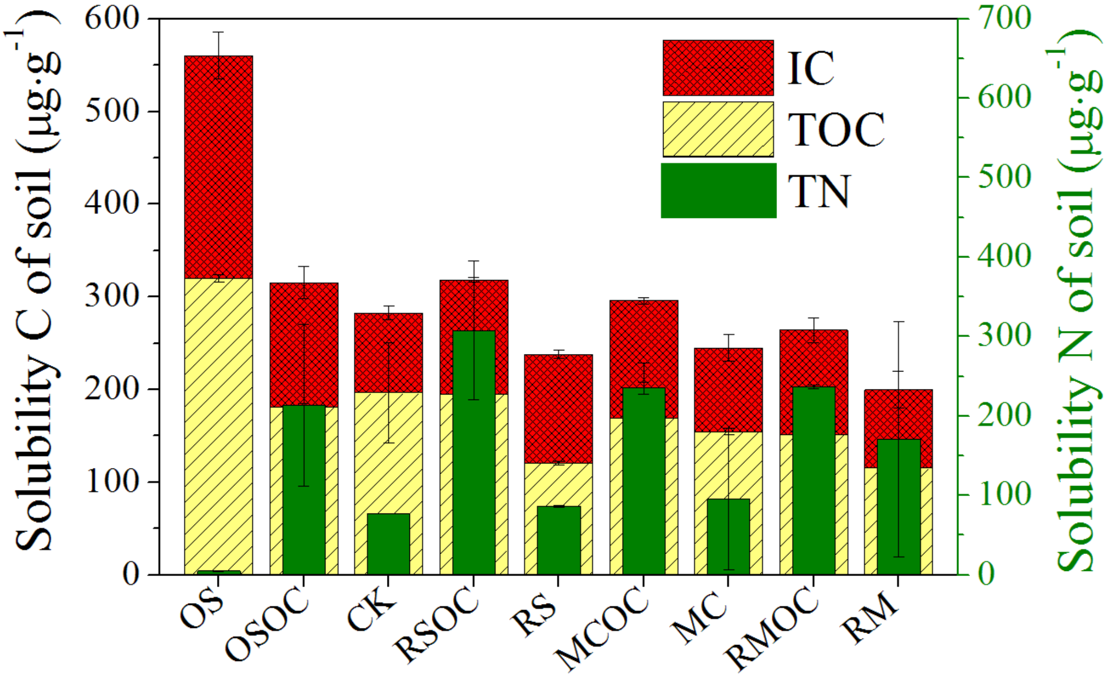

Figure S4 Contents for solubility total carbon (TC) and total nitrogen (TN) in soil samples. IC, inorganic carbon; TOC, total organic carbon; TC = IC + TOC.

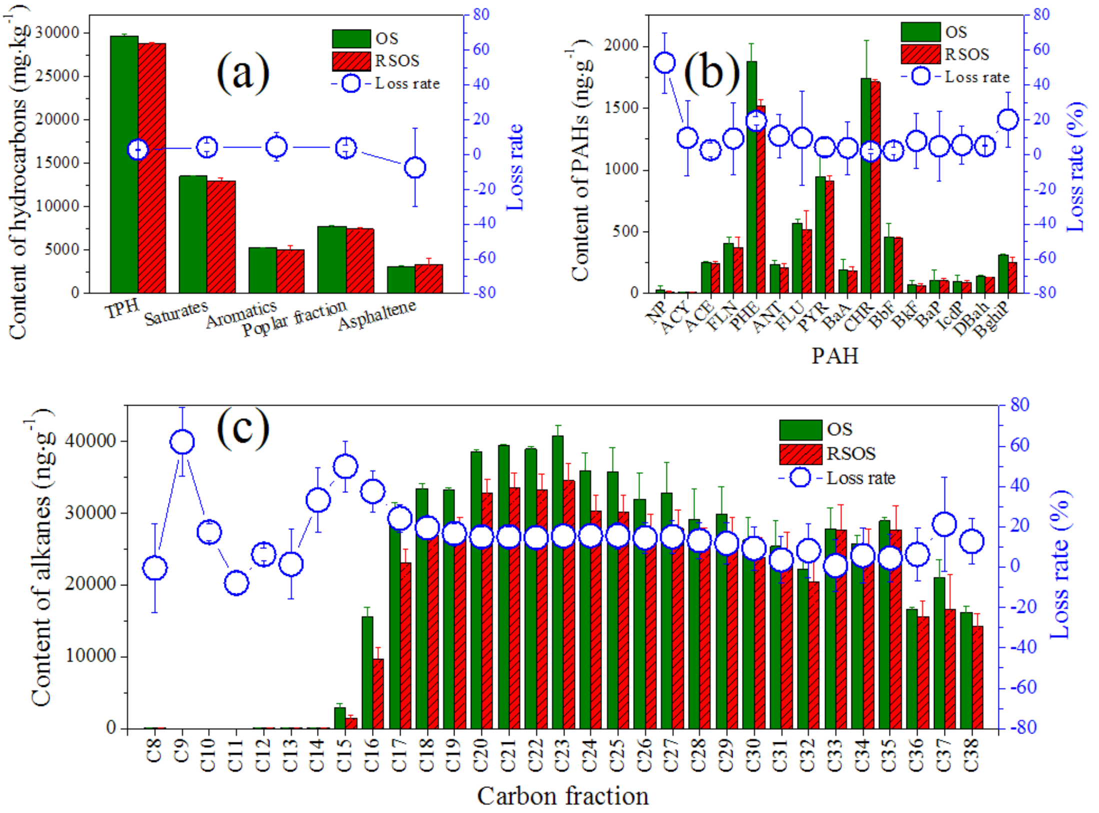

Figure S5 Contents for carbon fractions of petroleum hydrocarbons in original soil (OS) and soil after rinsed salt (RSOS). NP, naphthalene; ACY, acenaphthylene; ACE, acenaphthylene; FLN, fluorine; PHE, phenanthrene; ANT, anthracene; FLU, fluoranthene; PYR, pyrene; BaA, benzo(a)anthracene; CHR, chrysene; BbF, benzo(b)fluoranthene; BkF, benzo(k)fluoranthene; BaP, benzo(a)pyrene; IcdP, indeno(1,2,3-cd)pyrene; DBah, dibenzo(a,h)anthracene; BghiP, benzo(ghi)perylene. After rinsed salt, the loss rate of total petroleum hydrocarbons (TPHs) in RSOS was only 2.9% from OS, which was mainly determined by the saturates (4.2% of loss rate) and aromatics (4.5% of loss rate) rather than poplar fractions and asphaltene.

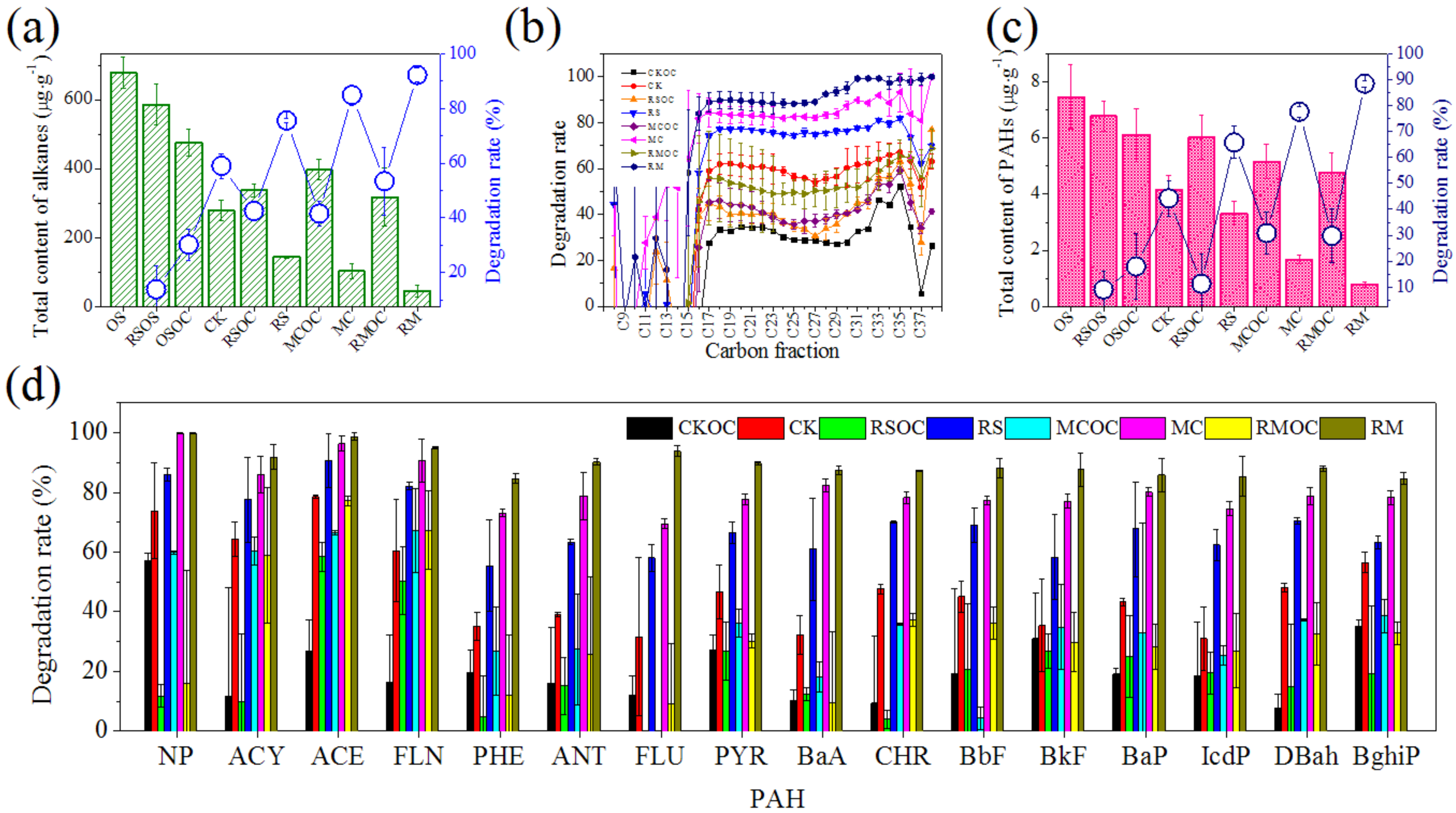

Figure S6 Contents and degradation rates for *n*-alkanes (a-b) and PAHs (c-d) in soil of MFCs.

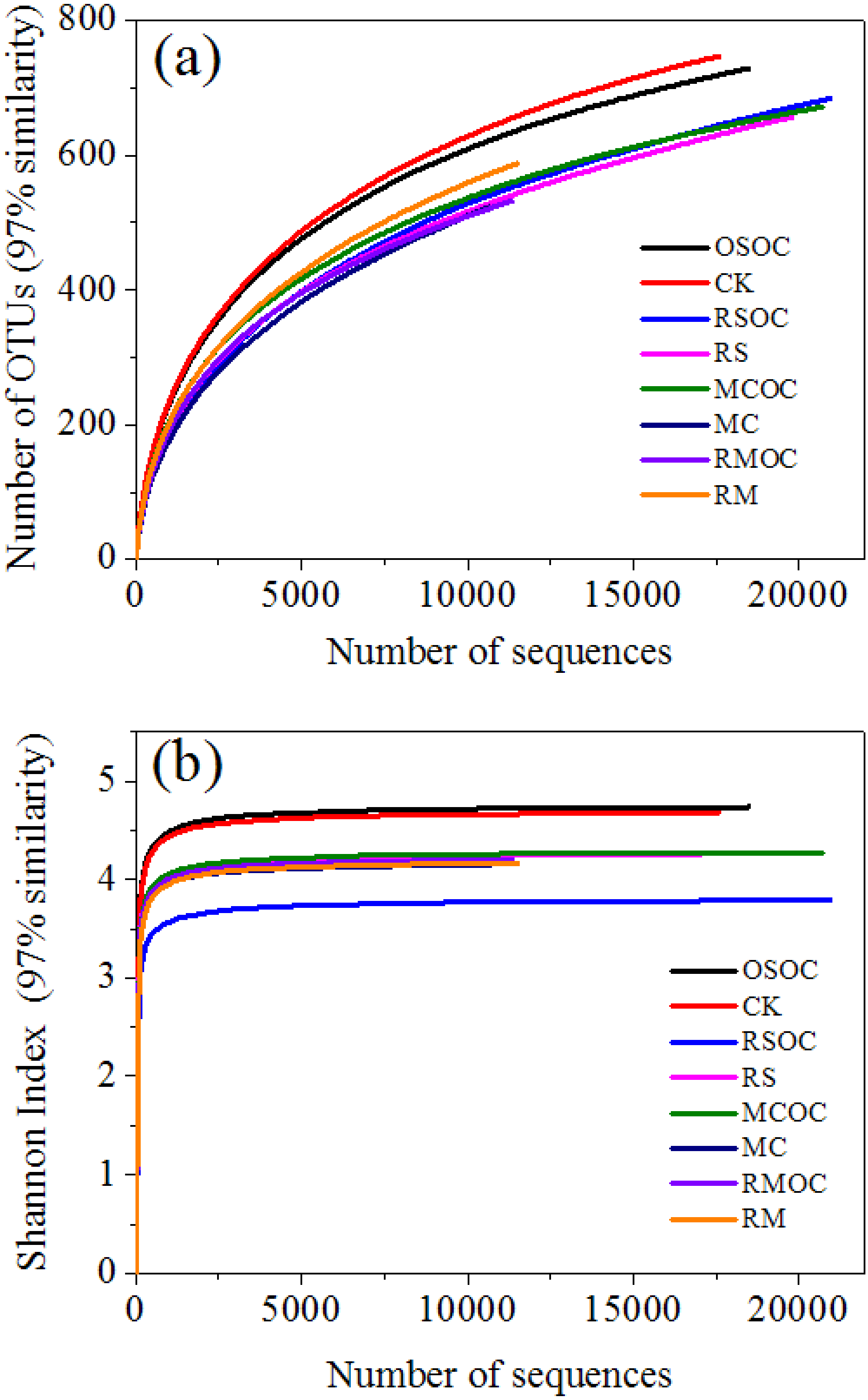

Figure S7 Rarefaction curves of the high-throughput sequencing for the soil samples.

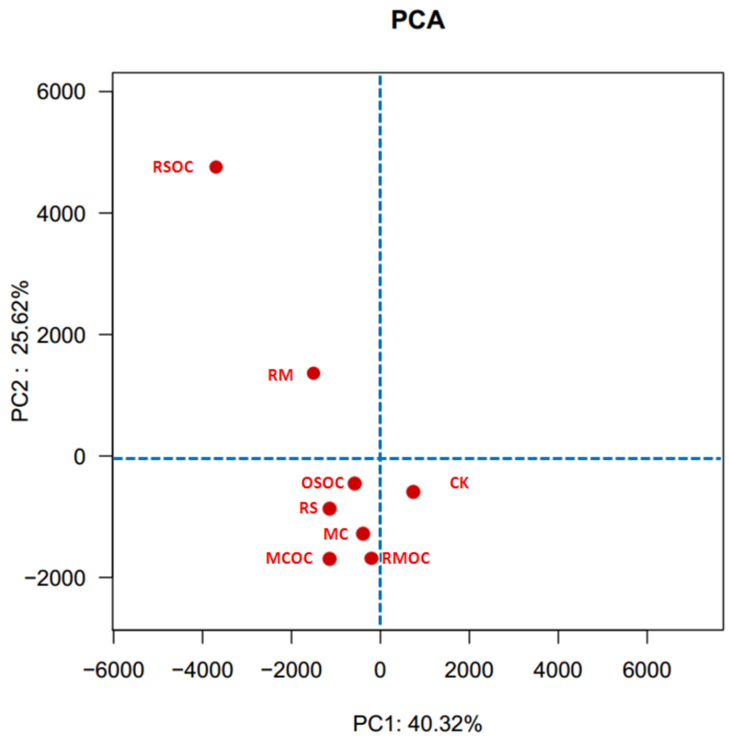

Figure S8 Principal component analysis (PCA) of bacterial communities based on pyrosequencing of 16S rRNA gene.

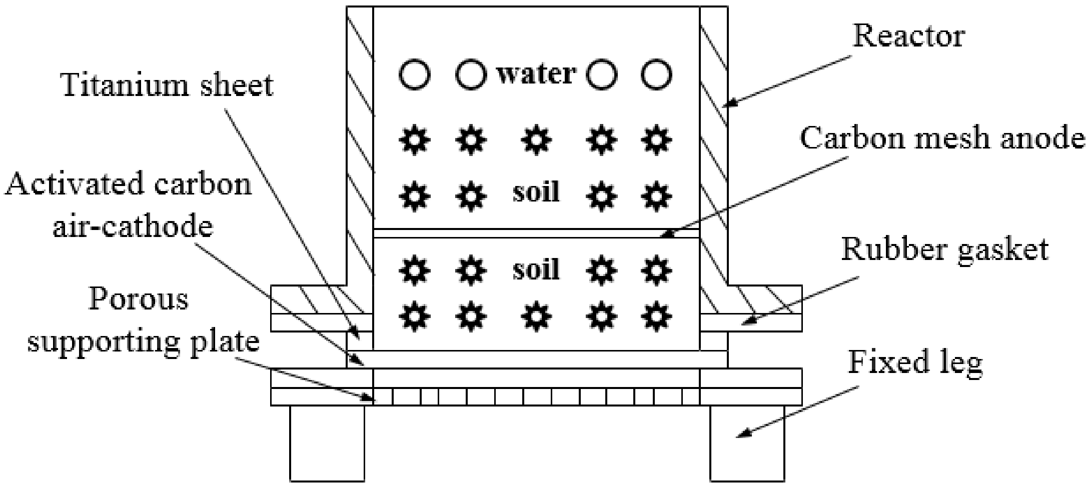

Figure S9 Schematics of a bioelectrochemical remediation system

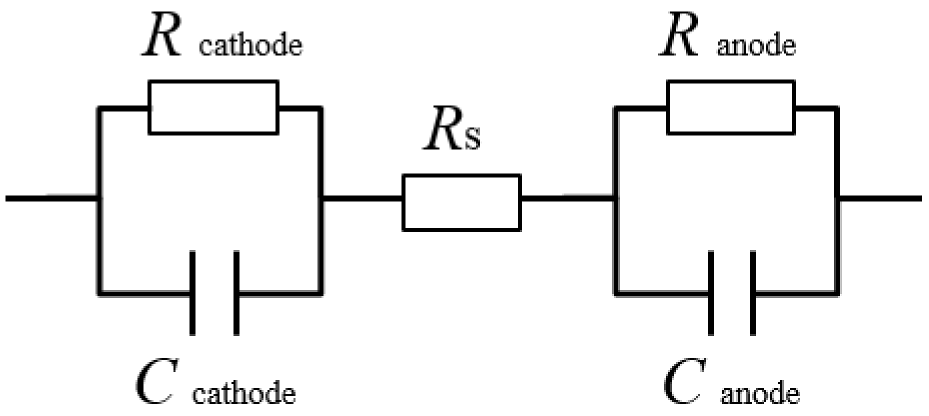

Figure S10 Equivalent circuit for modeling electrochemical impedance spectrum. *R*s indicates the Ohmic resistance of soil MFC.
